# Pembrolizumab-induced Stevens-Johnson syndrome^☆^

**DOI:** 10.1016/j.abd.2024.08.011

**Published:** 2025-04-10

**Authors:** Hiram Larangeira de Almeida Junior, Debora Sarzi Sartori, Aline Paganelli, Karen Francisca Borges Sias, Luísa Coelho Capuá, Darlan Cleverson Farezin

**Affiliations:** a Postgraduate Program in Health and Behavior, Universidade Católica de Pelotas, Pelotas, RS, Brazil; b Hospital School, Universidade Federal de Pelotas, Pelotas, RS, Brazil; c Laboratory Center for Pathological Anatomy, Pelotas, RS, Brazil; d Faculty of Medicine, Universidade Católica de Pelotas, Pelotas, RS, Brazil

*Dear Editor,*

Immunotherapy represents an important advance in the treatment of malignant neoplasms, and melanoma was the first disease for which its use was approved; over time it has gained other indications. Its therapeutic principle is the activation of T-lymphocytes, overcoming their inhibition with monoclonal antibodies targeted at proteins or receptors with an inhibitory effect.^1^ The 2018 Nobel Prize in Medicine was awarded to the discoverers of the possibility of releasing T-cells in oncological treatment, starting a new era.

In 2011, ipilimumab (an anti-CTLA-4 antibody) was approved for the treatment of metastatic melanoma, followed by the approval of nivolumab and pembrolizumab in 2014 (both anti-PD-1), all of them for melanoma.^1^ It was subsequently approved for other neoplasms, as well as combined therapy with two antibodies, with a synergistic effect, such as ipilimumab + nivolumab for melanoma in 2015.^1^

Its adverse effects are due to aggression of non-neoplastic tissues by activated T cells, the most common being thyroiditis (in 10% of patients receiving anti-PD-1 and up to 20% receiving combined therapy consisting of ipilimumab and nivolumab), hypophysitis (5% to 10% of patients; it is more common with ipilimumab) and adrenal insufficiency.^2^ Colitis, rheumatological conditions (resembling rheumatoid arthritis, polymyalgia rheumatica, polymyositis and Sjögren's syndrome), neurological and cardiac manifestations (myocarditis has a high mortality rate in these patients) are also described.

The skin may also be affected, with manifestations ranging from pruritus to exanthema, psoriasis, lichen planus, bullous pemphigoid, and even severe reactions in the Stevens-Johnson/Lyell syndrome spectrum.^2^, ^3^ Vitiligo is more common in patients treated for melanoma, suggesting immunity against neoplastic and epidermal melanocytes.^2^

The present report describes an 83-year-old female patient, previously hypertensive, with a history of thymoma excision in 2022. Two years later, she presented a vegetative lesion on the right heel, and histopathology revealed an undifferentiated malignant neoplasia, extending to the reticular dermis. Immunohistochemistry was positive for melanocytic markers, confirming the diagnosis of melanoma. Given the difficulty of the surgical approach, therapy with pembrolizumab was indicated by the oncology team. Ten days after she started treatment, she developed painful crusts in the oral cavity (Fig. 1A) associated with palmar lesions (Fig. 1C). As the condition progressed, target-like lesions with central erosion appeared (Fig. 1B).

**Fig. 1:**
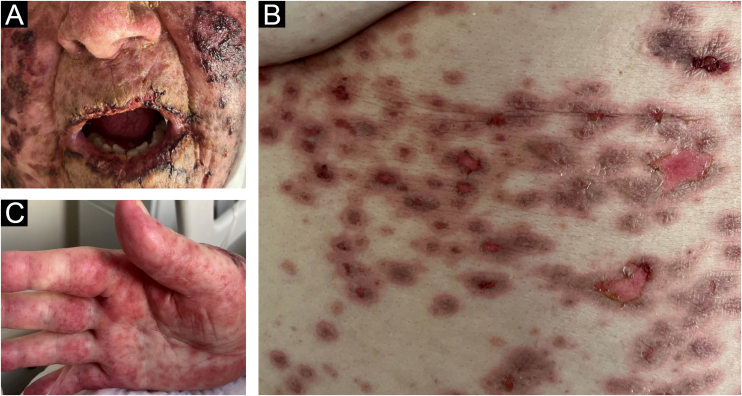
(A) Erosions on the labial mucosa and malar regions. (B) Palmar lesions. (C) Target-like lesions on the abdomen.

The patient was hospitalized and received systemic corticosteroid therapy. Despite the use of systemic antibiotics, the patient developed sepsis and died.

A biopsy of a target-like lesion was performed and histopathology revealed prominent necrosis of the epidermis with its detachment (Fig. 2A). A detailed examination identified epidermal necrosis in the area of ​​the bulla (Fig. 2B). In the transition to the unaffected area, there was necrosis of keratinocytes and the presence of numerous lymphocytes in the epidermis (Fig. 2C). On high magnification, several satellitosis figures were seen in this same area (Fig. 2D). The dermis exhibited scarce perivascular lymphocytic inflammatory infiltrate.

**Fig. 2:**
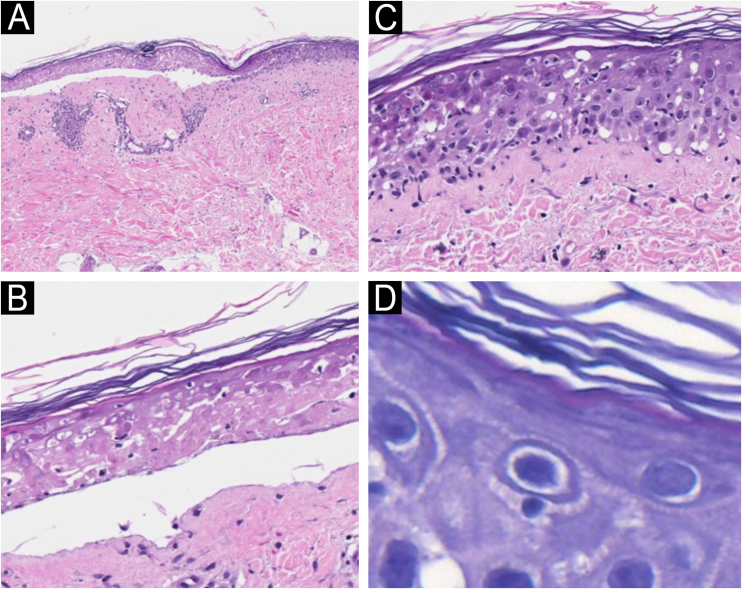
Light microscopy with hematoxylin & eosin. (A) Epidermal detachment and light inflammatory reaction in the dermis (×100). (B) Detail of epidermal necrosis in the center of the lesion (×200). (C) Detail of the periphery of the lesion with isolated necrosis of keratinocytes and lymphocytic exocytosis (×200). (D) Lymphocyte satellitosis at the periphery of the lesion (×400).

Immunohistochemistry (IHC) with anti-CD3, CD4, CD8 and CD20 antibodies, aimed to identify the lymphocyte subtypes involved, showed light dermal infiltrate of CD3, CD4 and CD8-positive cells; no positivity was seen for CD20 (Fig. 3). Detailed examination showed that the dermal infiltrate was predominantly composed by CD3 and CD4 positive cells and a smaller number of CD8 cells (Fig. 4). In the epidermis, the infiltrate was more pronounced with CD3-positive cells followed by CD8-positive cells (Fig. 4). The B lymphocyte marker, anti-CD20, was negative.

**Fig. 3:**
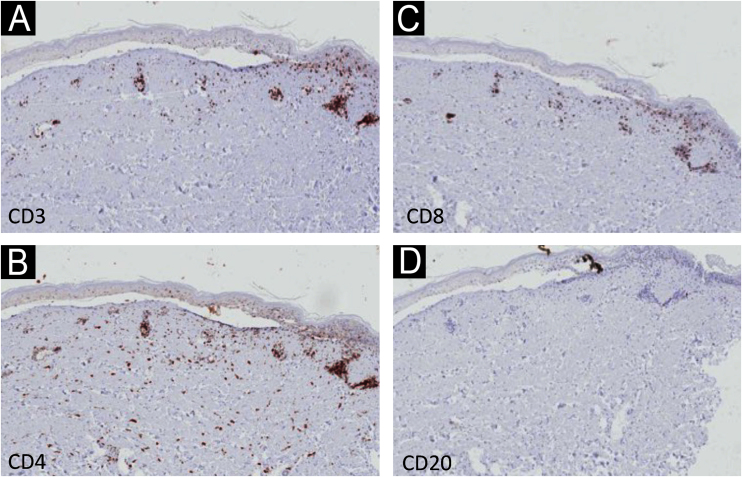
Immunohistochemistry – low magnification (×40) showing light dermal infiltrate with positivity for CD3, CD4 and CD8 cells (A‒C), and negativity for CD20 (D).

**Fig. 4:**
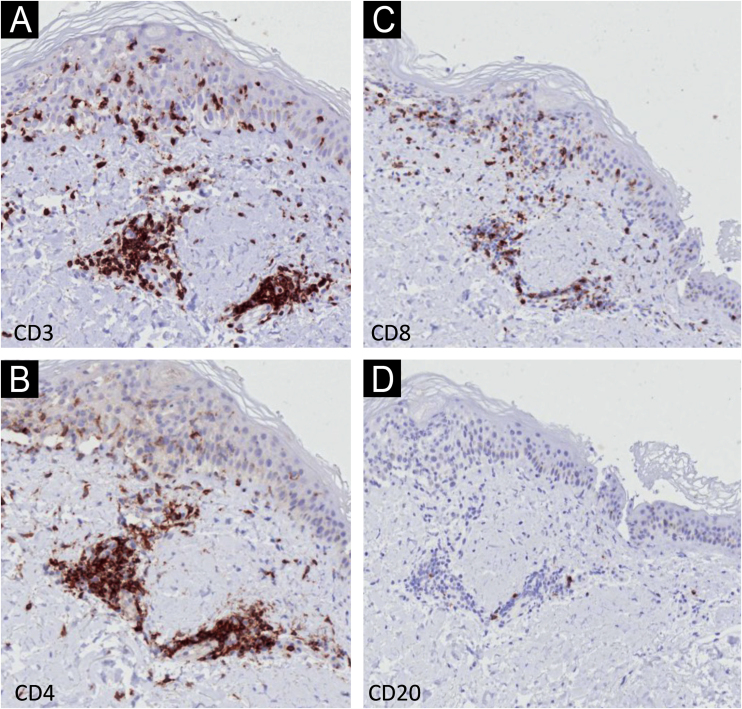
Immunohistochemistry – detail (×400) showing stronger positivity in the dermis for CD3 and CD4 (A and B) and in the epidermis for CD3 (A) and CD8 (C). Negativity for CD20 (D).

Given the clinical possibility of paraneoplastic pemphigus, indirect immunofluorescence was performed on rat bladder and normal skin, which resulted negative.

There are numerous reports of Stevens-Johnson syndrome associated with the use of pembrolizumab similarly to what was seen in the reported patient, sometimes after only one dose of immunotherapy,^4^, ^5^, ^6^, ^7^ but it can happen even after the ninth dose.^8^ There are also similar reports with other monoclonal antibodies used in immunotherapy.

The histopathological findings in the present report are similar to those previously described, with epidermal necrosis and little dermal infiltration.^9^ There is only one IHC report demonstrating a predominance of CD8 cells, in slight disagreement with the present findings of epidermal predominance of CD3 cells over CD8-positive cells; the CD3 protein participates in the activation of CD8-positive cells, and their co-expression may occur.^9^ It is necessary to examine more cases with IHC to better understand these findings.

The satellitosis found in the present case is characteristic of T-cell-mediated diseases, such as graft-versus-host disease, and demonstrates the activation of T cells by immunotherapy.^10^

The IHC findings confirm epidermal aggression by T-lineage cells.

The literature on pembrolizumab-induced Stevens-Johnson syndrome refers to different neoplasms, outcomes, and intensities, including an association with other autoimmune diseases,^7^, ^8^ which may contribute to the severity of this condition, and therefore, dermatologists should be aware of these possibilities.

## Financial support

None declared.

## Authors' contributions

Hiram Larangeira de Almeida Jr: Approval of the final version of the manuscript; design and planning of the study; drafting and editing of the manuscript; collection, analysis and interpretation of data; effective participation in research orientation; intellectual participation in the propaedeutic and/or therapeutic conduct of the studied cases; critical review of the literature; critical review of the manuscript.

Debora Sarzi Sartori: Approval of the final version of the manuscript; design and planning of the study; drafting and editing of the manuscript; collection, analysis and interpretation of data; intellectual participation in the propaedeutic and/or therapeutic conduct of the studied cases; critical review of the literature; critical review of the manuscript.

Aline Paganelli: Approval of the final version of the manuscript; design and planning of the study; drafting and editing of the manuscript; collection, analysis and interpretation of data; effective participation in research orientation; critical review of the literature; critical review of the manuscript.

Karen Francisca Borges Sias: Approval of the final version of the manuscript; drafting and editing of the manuscript; collection, analysis and interpretation of data; intellectual participation in the propaedeutic and/or therapeutic conduct of the studied cases; critical review of the literature; critical review of the manuscript.

Luísa Coelho Capuá: Approval of the final version of the manuscript; drafting and editing of the manuscript; collection, analysis and interpretation of data; intellectual participation in the propaedeutic and/or therapeutic conduct of the studied cases; critical review of the literature; critical review of the manuscript.

Darlan Cleerson Farezin: Approval of the final version of the manuscript; drafting and editing of the manuscript; collection, analysis and interpretation of data; intellectual participation in the propaedeutic and/or therapeutic conduct of the studied cases; critical review of the literature; critical review of the manuscript.

## Conflicts of interest

None declared.
